# 1855. Risk factors for early mortality in patients with carbapenem-resistant *Acinetobacter baumannii* bacteremia

**DOI:** 10.1093/ofid/ofac492.1484

**Published:** 2022-12-15

**Authors:** Chan Mi Lee, Chung-Jong Kim, Seong Eun Kim, Kyung-Hwa Park, Ji Yun Bae, Hee Jung Choi, YoungHee Jung, Seung Soon Lee, Pyoeng Gyun Choe, Wan Beom Park, Eu Suk Kim, Je Eun Song, Yee Gyung Kwak, Sun Hee Lee, Shinwon Lee, Shinhye Cheon, Yeon Sook Kim, Yu Min Kang, Ji Hwan Bang, Sook-In Jung, Kyoung-Ho Song, Hong Bin Kim

**Affiliations:** Seoul National University College of Medicine, Seoul, Seoul-t'ukpyolsi, Republic of Korea; Ewha Womans University College of Medicine, Seoul, Seoul-t'ukpyolsi, Republic of Korea; Chonnam National University Medical School, Gwangju, Kwangju-jikhalsi, Republic of Korea; Chonnam National University Medical School, Gwangju, Kwangju-jikhalsi, Republic of Korea; Ewha Womans University College of Medicine, Seoul, Seoul-t'ukpyolsi, Republic of Korea; Ewha Womans University College of Medicine, Seoul, Seoul-t'ukpyolsi, Republic of Korea; Seoul National University College of Medicine, Seoul, Seoul-t'ukpyolsi, Republic of Korea; Hallym University Chuncheon Sacred Heart Hospital, Chuncheon, Kangwon-do, Republic of Korea; Seoul National University College of Medicine, Seoul, Seoul-t'ukpyolsi, Republic of Korea; Seoul National University College of Medicine, Seoul, Seoul-t'ukpyolsi, Republic of Korea; Seoul National University College of Medicine, Seoul, Seoul-t'ukpyolsi, Republic of Korea; Inje University Ilsan Paik Hospital, Goyang, Kyonggi-do, Republic of Korea; Ilsan Paik Hospital, Ilsan, Kyonggi-do, Republic of Korea; Division of Infectious Disease, Department of Internal Medicine, Pusan National University Hospital, Seo-gu, Pusan-jikhalsi, Republic of Korea; Division of Infectious Disease, Department of Internal Medicine, Pusan National University Hospital, Seo-gu, Pusan-jikhalsi, Republic of Korea; Chungnam National University Hospital, Daejeon, Ch'ungch'ong-namdo, Republic of Korea; Chungnam National University School of Medicine, Daejeon, Ch'ungch'ong-namdo, Republic of Korea; Myongji Hospital, Goyang, Kyonggi-do, Republic of Korea; Seoul National University College of Medicine, Seoul, Seoul-t'ukpyolsi, Republic of Korea; Chonnam National University Medical School, Gwangju, Kwangju-jikhalsi, Republic of Korea; Seoul National University College of Medicine, Seoul, Seoul-t'ukpyolsi, Republic of Korea; Seoul National University College of Medicine, Seoul, Seoul-t'ukpyolsi, Republic of Korea

## Abstract

**Background:**

Although many deaths due to carbapenem-resistant *Acinetobacter baumannii* (CRAB) bacteremia occur within a few days after the onset of bacteremia, risk factors for early mortality (EM) have not been deeply investigated. We aimed to determine the risk factors for EM and the difference between risk factors associated with EM and late mortality (LM) in CRAB bacteremia.

**Methods:**

All patients with CRAB bacteremia in 10 hospitals during a 1-year study period were identified. We prospectively collected patients’ clinical data, including microbiological and demographic data, underlying comorbidities, origin of bacteremia, severity of illness, antibiotic therapy, and mortality. Among the cases with mortality within 30 days, EM and LM were defined as death within 3 and more than 5 calendar days from the first positive blood culture, respectively.

**Results:**

A total of 212 CRAB bacteremia cases were included in the analysis. Of 122 (57.5%) patients with 30-day mortality, EM was observed in 75 (61.5%) patients and LM in 39 (32.0%) patients. The proportion of severe sepsis or septic shock, Pitt score, and Sequential Organ Failure Assessment (SOFA) score were significantly higher in 30-day deaths than 30-day survivors. These factors of clinical severity were also significantly higher in patients with EM than those with LM. While urinary tract infection as the factor of site of infection and the severity of illness were independent predictors of LM, only factors representing the severity of illness were independent risk factors for EM. Appropriate empirical antibiotic therapy was associated with reduced risk of EM.

**Figure:**
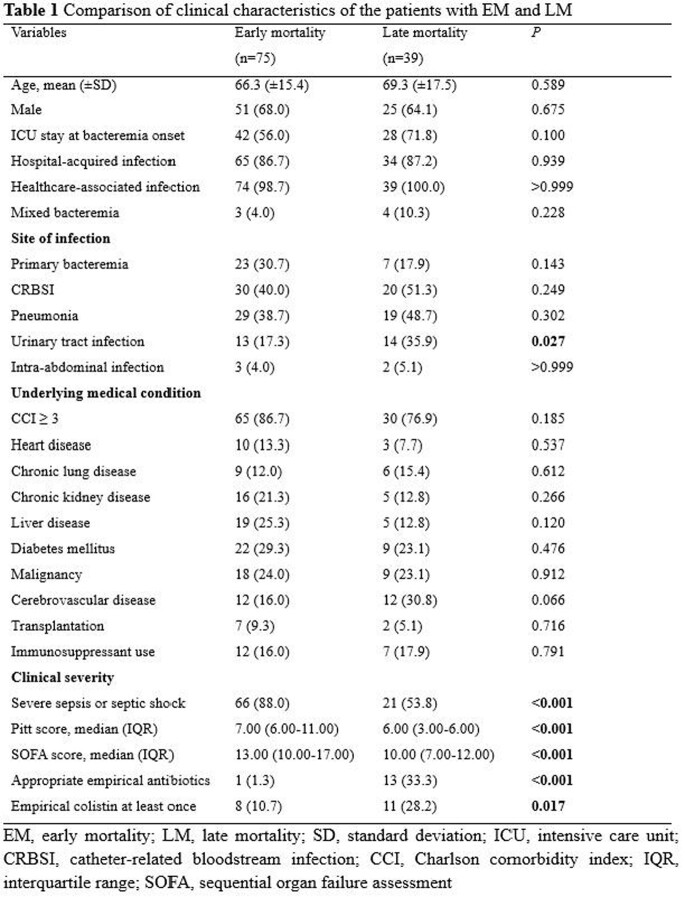

**Figure:**
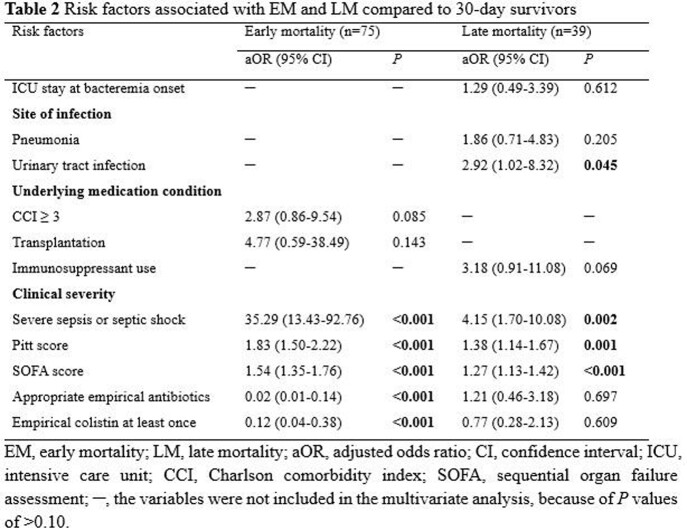

**Conclusion:**

The difference between risk factors for EM and LM was identified in this study. Our data suggest that a large proportion of CRAB bacteremia with high severity progress to a rapidly fatal course, regardless of the underlying diseases or source of infection. Further studies might be needed to investigate the microbiological factors associated with CRAB and pathogen-host interaction in patients with EM.

**Disclosures:**

**All Authors**: No reported disclosures.

